# Hernia of Morgagni Presenting as Constipation in a 65-Year-Old Male

**DOI:** 10.7759/cureus.1278

**Published:** 2017-05-26

**Authors:** Hamza A Khan, Adeel Samad, Omar A Khan, Farida K Chagan, Jamal K Khan, Saulat H Fatimi

**Affiliations:** 1 Medical College, The Aga Khan University; 2 Medical College, Jinnah Sindh Medical University (SMC); 3 Department of Surgery, The Aga Khan University; 4 Department of Cardiothoracic Surgery, The Aga Khan University

**Keywords:** morgagni, hernia, diaphragm, constipation

## Abstract

Hernia of Morgagni is congenital defects in the diaphragm. They are mostly asymptomatic and present with vague symptoms when they do so. A high index of suspicion is required for timely diagnose of this condition. Here we present the case of a 65-year-old male patient presented to our institute with constipation for the past six months. Chest radiology raised the suspicion of a hernia which was further confirmed by contrast studies. Laparotomy was done and the hernia sac identified, colon and greater omentum reduced and defect repaired. He was discharged in stable condition and was doing well on follow-up.

## Introduction

Hernia of Morgagni (MH) is a congenital defect in the diaphragm. It is located posterolateral to the sternum and is also referred as a retrosternal, parasternal or subcostosternal diaphragmatic hernia. Giovanni Morgagni first described it in 1761 while performing a post-mortem examination and hence the name. It is the rarest of all congenital diaphragmatic hernias (CDH) accounting for only 2%-3% of all cases [1-2]. CDH occurs in one out of two-3,000 live births, accounting for 8% of all congenital anomalies [3]. Bochdalek hernia, hiatus hernia, and anterior Morgagni hernia are the basic types of CDH.

These hernias are more common in the pediatric population than in the adult. The foramina of Morgagni is a retrosternal and anterior defect in the diaphragm and these hernias usually occur in the anterior mediastinum or on the right side. MH frequently contain the omental fat accompanied by the transverse colon. Horton, et al. reports 91% right-sided cases in his study with predisposing factors in 41% of them. Females constituted 62% of the cases with the average age of the sample being 53 years of age [4]. Informed consent statement was obtained for this study.

## Case presentation

A 65- year-old male with past medical history of hypertension presented to the hospital with a six-month history of severe constipation intermittently. He had a bowel movement every two to three days. He had tried multiple medications and herbal treatment and at presentation required enemas to relieve his constipation. He, however, was passing flatus and had no other associated symptoms. He denied any abdominal pain, weight loss, change in stool or any significant family history. He reported a balanced diet. The patient was a laborer by profession. 

On physical examination, at the time of presentation in the outpatient clinic, he did not show any signs of intestinal obstruction although reduced air entry was noticed bilaterally in the lower lung fields. His general physical examination and other systemic examinations were unremarkable.

**Figure 1:**
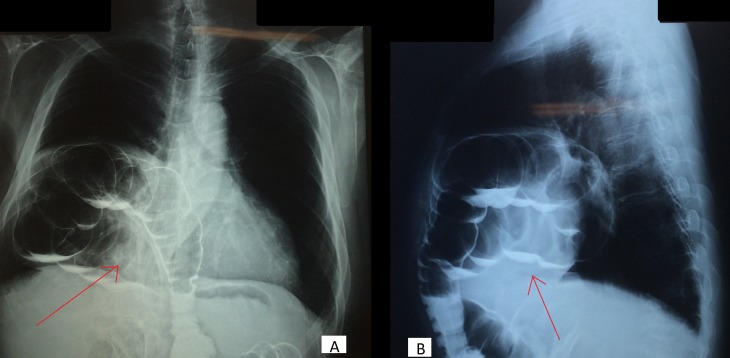
A: Anterio-posterior and B: Lateral view- Barium enema with arrows showing bowel herniating into the thoracic cavity

The patient has advised baselines labs along with chest radiology and barium study. His baseline labs that included complete blood count turned to be normal. Chest radiography raised the suspicion of bowel in the pleural cavity. His barium study, however, confirmed the diagnosis as stated below (Figure 1).

 

**Figure 2:**
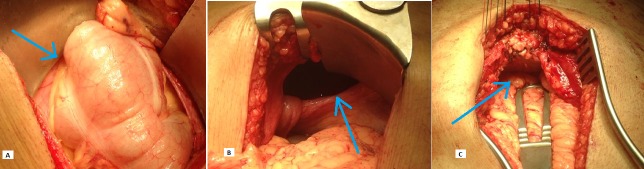
Contents of Morgagni hernia seen in 'A' (arrow) through a defect in diaphragm visible in 'B' (arrow) and repair of the defect seen in 'C' (arrow)

The patient was admitted for surgical closure of a hernia. An upper central abdominal incision was given and upon opening the peritoneal cavity, the defect in the diaphragm was clearly visualized (Figure 2) along with the hernia sac and its contents, which were colon and greater omentum. The sac was opened and the contents were reduced back into the peritoneal cavity. Primary repair of the anterior defect in the diaphragm was carried out with interrupted ethibond 2/0 pledgeted sutures to the anterior abdominal wall, which was further reinforced with zero polypropylene suture.

Postoperatively, the patient was mobilized, his diet progressed and was discharged once he was stable. He followed up in clinic after a few weeks and was doing well with regular bowel movements. Last follow-up after six months was unremarkable.

## Discussion

Congenital Morgagni hernia is believed to be caused by a failure in the fusion of the septum transverse of the diaphragm and the costal arches, which later on exacerbated by increased intraperitoneal pressure gives rise to the hernia [5]. Pathophysiology of Morgagni hernia in adults is still unknown owing to the rarity of the disease. Whether the disease is congenital in origin or acquired is still under consideration. It is presumed that predisposing conditions that increase intra-abdominal pressure like a chronic cough, pregnancy, chronic constipation and trauma act on the pre-existing diaphragmatic defect in causing the hernia [4]. This theory is supported by patients with Morgagni hernia who initially had unremarkable radiographs [6].

It presents with non-specific symptoms in adults such as vague abdominal discomfort, flatulence, indigestion, constipation, loss of appetite or even respiratory compromise. However, cases with bowel obstruction and strangulation have also been reported. Some patients can even be asymptomatic and diagnosed only incidentally. Since these symptoms are so common, often they have attributed to other factors such as dyspepsia that is missed. A high index of suspicion is therefore required. Neonates present with acute abdomen or acute respiratory distress syndrome whereas children might present with repeated chest infections [7].

X-ray radiographs are effective diagnostic tools for Morgagni hernia which appear as fatty mass, if the omental fat is herniating or as bowel loops if it’s along with the large intestine [6]. Prominent epicardial fat pads, lipomas, sarcomas, thymomas and even pulmonary lesions like pulmonary tuberculosis can give a similar appearance on x-ray. In these circumstances, other tools like ultrasonography and computed tomography (CT) is considered. Contrast studies such as barium enema become less reliable in cases where only the omental fat or part of the liver is herniated through the defect [4]. Currently, CT is the most sensitive of all these modalities giving anatomical details and complications. Magnetic resonance imaging (MRI) is equally reliable but expense leads to it being used only when contrast is contraindicated. However, in some cases where the bowel keeps sliding in and out of the defect, even modalities like CT and MRI prove difficult and a diagnostic laparoscopy is indicated [6].

Today Morgagni hernias are no longer considered emergency cases, however, each patient is evaluated individually and surgical management is recommended to avoid any emergencies. If the defect is larger or the bowel is in the sac, the risk of herniation becomes high and surgery is indicated. With older patients, general anesthesia and surgical risks are also given special attention. Kuster, et al. reported the first laparoscopic repair in 1992 following which laparoscopy has become the mainstay [8]. It has been proven to be safe with faster recovery, less trauma and faster return to normal function. Trans-abdominal and trans-thoracic are the two approaches known. The abdominal approach is preferred unless the diagnosis is uncertain. Recurrent rates of 2%-15% postoperatively are reported due to the remnant pouch or otherwise [9]. Only recently has Arevalo, et al. reported successful minimally invasive management of Morgagni hernia with robotic use [10].

## Conclusions

In conclusion, we presented a rare case of a hernia of Morgagni in an elderly male presenting as constipation. Even though they are no longer regarded as emergency cases, surgical correction must be recommended to avoid future emergencies where bowel can herniate into the sac. Benign presentations are rare and hence a high index of suspicion is required.
